# The Role of Cold Rolling Reduction on the Microstructure and Mechanical Properties of Ultra-Low Carbon Bainitic Steel

**DOI:** 10.3390/ma15093070

**Published:** 2022-04-23

**Authors:** Zemin Wang, Yu Dong, Jiajun Li, Feng Chai, Lianbo Wang, Qingdong Liu, Bin Fu, Min Liu, Zhanyong Wang

**Affiliations:** 1 School of Materials Science and Engineering, Shanghai Institute of Technology, Shanghai 201418, China; 196082113@mail.sit.edu.cn (Y.D.); peughostrip@163.com (J.L.); wanglianbo021@hotmail.com (L.W.); fubin@sit.edu.cn (B.F.); liumin110ok@163.com (M.L.); zhanyong.wang@vip.sina.com (Z.W.); 2 Institute of Engineering Steel, Central Iron & Steel Research Institute, Beijing 100081, China; 3 Collaborative Innovation Center for Advanced Ship and Deep-Sea Exploration, Shanghai Jiaotong University, Shanghai 200240, China; qdliu@sjtu.edu.cn

**Keywords:** ultra-low carbon bainitic steel, cold rolling, high strength, dislocation density, mechanical properties

## Abstract

The present study investigates the microstructure and mechanical properties of ultra-low carbon bainitic steel (UCBS) under different cold rolling reductions. When the rolling reduction ratios were increased to 80%, the microstructure was refined, and the lath width of the bainite decreased from 601 nm to 252 nm. The ultimate tensile strength and yield strength increased from 812 MPa and 683 MPa to 1195 MPa and 1150 MPa, respectively, whereas the elongation decreased from 15.9% to 7.9%. In addition, the dislocation density increased from 8.3 × 10^13^ m^−2^ to 4.87 × 10^14^ m^−2^ and a stronger γ-fiber texture was obtained at the 80% cold rolling reduction ratio. The local stress distribution and kernel average misorientation were not uniform and became more severe with increased rolling reduction ratios. The strength increment of UCBS was primarily due to boundary strengthening and dislocation strengthening. The theoretical strength increment agreed well with the experimental measurements, which can be helpful for the design and production of UCBS for broad engineering applications.

## 1. Introduction

Ultra-low carbon bainitic steel (UCBS) is widely used in ship manufacturing, offshore oil platforms, bridge construction, and aviation facilities due to its excellent strength, high ductility, good weldability, and low cost [1,2,3,4]. In previous studies, high strength UCBS was mainly obtained by controlling the microstructure through different methods, such as grain refinement [5,6], precipitation hardening during annealing (e.g., Nb, V, Mo, Ti-contained carbides, Cu-rich precipitates) [3,7,8,9] and deformation strengthening (i.e., shape controlling) [5,8,10,11].

In order to expand its application for certain products, UCBS is often cold-rolled into strips [12]. Nevertheless, the cold rolling reduction has significant effects on the microstructure and mechanical properties of UCBS. In particular, the microstructure can be severely fragmented. The high dislocation density and anisotropy are introduced to store energy [12,13,14,15]. It has been reported that the hardness and strength increased, while the ductility decreased as the deformation increased, which is attributed to dislocation accumulation and boundary strength (grain size decreases) [16,17,18,19,20,21]. However, for deformed UCBS, the grains are stretched and paralleled to the rolling direction. Then, it is relatively weak at predicting yield stress and grain size by the Hall–Petch relation, especially for grain size smaller than 20 nm. Swarr et al. [22], Wang et al. [23], and Luo et al. [24] reported that the width of both the martensitic lath and block determined the mechanical properties, which follows the Hall–Petch relation. However, little attention has been paid to quantitatively assessing the dislocation density and boundary strength in UCBS during the cold rolling process.

Meanwhile, it has also been found that the weak texture of Fe-0.025% C steel gradually changes from ζ-fiber in the as-received to γ and θ-fiber after cold rolling [25]. On the other hand, it has been reported that, after a 90% rolling reduction in Fe-0.82% C steel, only the θ-fiber texture of ferrite was obtained [26]. The effect of the microstructure of cold-rolled steel on deformation behaviors and the development of crystallography constitutes in UCBS have not yet been fully explored.

In the present study, X-ray diffraction (XRD), transmission electron microscopy (TEM), and electron back scattered diffraction (EBSD) techniques are utilized to investigate the microstructure and mechanical properties of UCBS under different cold rolling reductions. The results showed the contribution of two strengthening mechanisms and the development of crystallography under different cold rolling reductions. Finally, the strength increments are also experimentally and theoretically determined.

## 2. Materials and Methods

### 2.1. Material Preparation

The nominal chemical composition of the investigated steel was Fe- 0.04C- 2.35Ni- 1.45Cu- 0.9Cr- 0.41Mo (in wt.%). The ingots were molten in a vacuum induction furnace. Steel sheets with a thickness of 10 mm were produced through controlled thermomechanical treatments. The thermal expansion data of the steel was measured by the NETZSCH thermal dilatometer. The austenitic transition starting temperature (Ac1) and finishing temperature (Ac3) were about 703 °C and 852 °C, respectively (the heating rate is 5 °C/min). The sheets were treated with the solid solution at 900 °C for 1 h, the typical lath microstructure was obtained after water quenching. Subsequently, the sheets were cold rolled in several passes (1 mm/passes) to final thicknesses of 8 mm, 5 mm, and 2 mm at ambient temperature; each pass corresponds to deformation ratios of 20%, 50%, and 80%, respectively.

### 2.2. Materials Characterization

**Figure 1:**
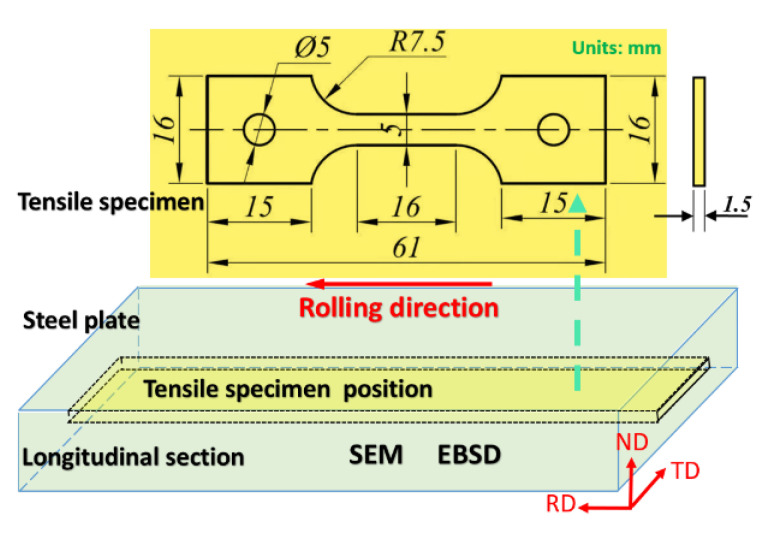
Schematic diagram of SEM, EBSD observation, and tensile specimen.

The samples for scanning electron microscopy (SEM) observation were mechanically ground and polished and then etched using 4 vol% nitric acid in alcohol. After mechanical grinding and polishing, samples for EBSD were prepared by electro-polishing using 5 vol% perchloric acid in alcohol. SEM and EBSD observations (RD-ND section in Figure 1) were conducted on a JEM-6700 and FEI SEM, respectively. The raw data were analyzed in the TSL-OIM-Analysis software.

The samples for TEM were mechanically ground to 50 μm thickness, and subsequently electro-polished by a twin-jet Struer Tenupol-2 using 10 vol% perchloric acid in alcohol at −30 °C and 30 V. The width of lath bainte were measured by TEM on a Tecnai G2 F20 S-TWIN field emission gun (FEG) TEM instrument operating at 200 kV.

The samples for crystal analysis (RD-TD section in Figure 1) were examined by the Rigaku XRD instrument after electro-polishing with Cu-Kα irradiation (40 kV, 30 mA, λ = 0.1541 nm). Finally, θ-2θ scanning was conducted in the range of 30° to 90° at a speed of 2° min^−1^ and step size of 0.02° [18]. In order to quantitatively evaluate the dislocation density by XRD, a reference sample was prepared after annealing at 1000 °C for 12 h.

### 2.3. Mechanical Properties Measurement

The microhardness was determined from metallographic polished specimens by a 402SXV SCTMC microhardness tester with a loading of 200 g and dwell time of 5 s. In order to evaluate the mechanical properties, 16 mm (width) × 1.5 mm (thickness) × 61 mm (length) specimens were machined. The location and dimensional drawing of the tensile specimen is shown in Figure 1. The tensile tests were performed by a tensile machine (Zwick/Roell Z100) at a displacement rate of 0.2 mm/min at ambient temperature.

## 3. Results

### 3.1. Mechanical Properties

**Figure 2:**
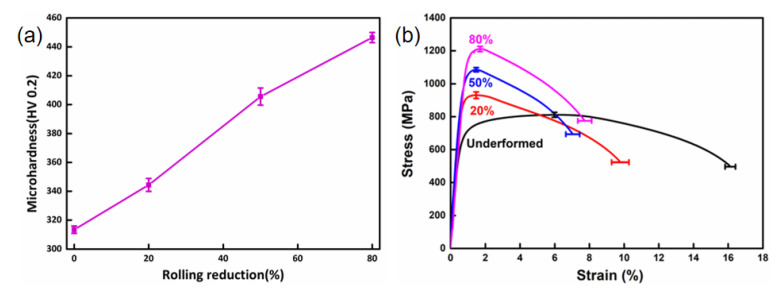
Variation of mechanical properties of UCBS in different cold rolling reductions. (**a**) Microhardness, (**b**) Tensile stress-strain curves.

The variation of the mechanical properties of UCBS under different cold rolling reductions is presented in Figure 2. With the cold rolling reduction ratios increased from 0 to 80%, the hardness increased dramatically from 313 HV to 446 HV (Figure 2a). Similarly, the ultimate tensile strength (UTS) and yield strength (YS) values increased from 812 MPa and 683 MPa to 1195 MPa and 1150 MPa, respectively (Figure 2b). The elongation was decreased from 15.9% to 7.9%. Schindler [27] and Liu [28] have also reported that the strength of UCBS steel gradually increased, and its ductility decreased with the increasing cold rolling reduction ratio. This increase in strength and decrease in plasticity can be attributed to the dislocation interaction and boundary strengthening [11,13,29,30].

### 3.2. Microstructure

**Figure 3:**
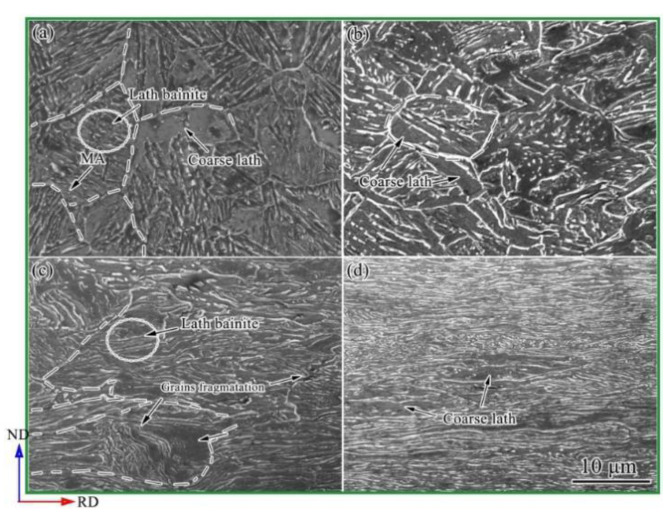
SEM microstructure images of UCBS. (**a**) undeformed; (**b**) 20%, (**c**) 50%, and (**d**) 80% cold rolling reduction ratio.

Figure 3a reveals typical lath bainite in a quench sample, with an average grain size of ~15 μm, as well as a small amount of martensite/austenite island (M/A) [31]. The lath direction was randomly distributed. When the rolling reduction ratio increased from 20% to 80%, the grain shape was elongated along the rolling direction (Figure 3b–d). The equiaxed grains were gradually refined and fragmented. Furthermore, the amount of MA decreased with the rolling reduction ratio increasing, which might be the deformation-induced ferrite transformation during cold rolling.

**Figure 4:**
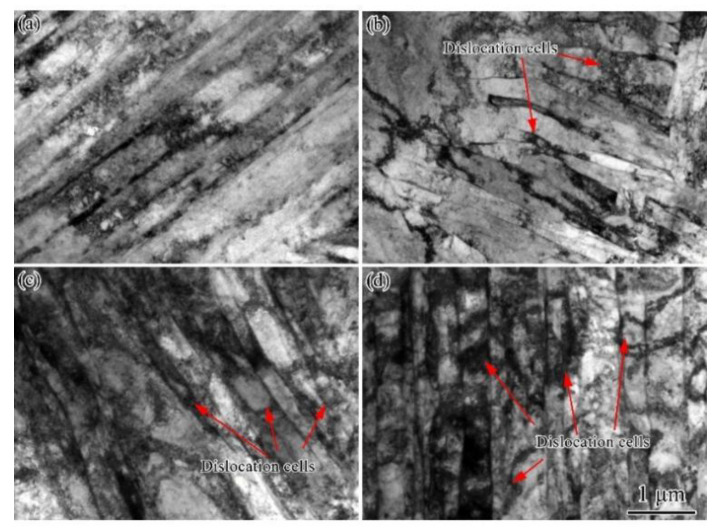
TEM images of UCBS obtained under different cold rolling reductions. (**a**) undeformed; (**b**) 20%, (**c**) 50%, and (**d**) 80% rolling reduction ratio.

Figure 4 depicts the TEM micrographs of UCBS obtained under different cold rolling reductions. It can be observed that the microstructure in all conditions consisted predominantly of laths. In comparison, the undeformed steel exhibited a low dislocation density (Figure 4a). As shown in Figure 4b–d, the dislocation density gradually increased with the increasing cold rolling reduction, corresponding to increased microhardness and strength. In order to accurately measure the thickness of laths by the intercept method [32,33], the average thickness of laths was measured from 150 laths in three TEM samples at the same condition. The thickness of laths decreased gradually from 601 ± 32 nm in the undeformed to 404 ± 18 nm, 320 ± 25 nm, and 252 ±16 nm, after 20%, 50%, and 80% cold rolling reduction ratio, respectively.

### 3.3. EBSD Observations

**Figure 5:**
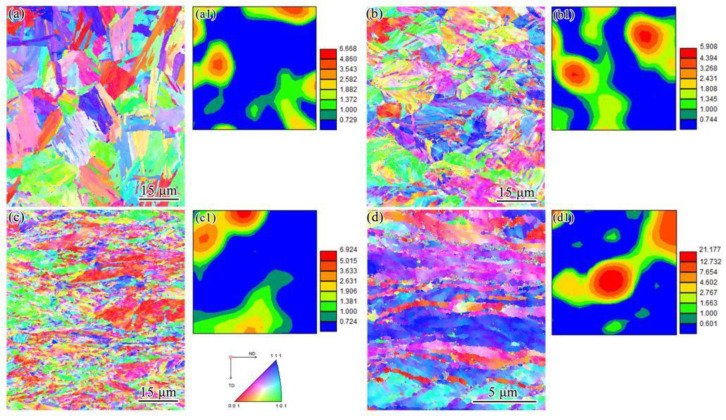
IPF maps at a cross-section in the TD–ND planes of UCBS. (**a**) undeformed; rolling reduction of (**b**) 20%, (**c**) 50%, and (**d**) 80%; ODF image in a φ2 = 45° section of (**a1**) undeformed sample, and deformed samples under a rolling reduction ratio of (**b2**) 20%, (**c1**) 50%, and (**d1**) 80%.

Figure 5 exhibits the inverse pole figure (IPF) maps and orientation distribution functions (ODF) in the transverse direction–normal direction (TD–ND) planes of UCBS samples obtained under different reductions. The red, blue, and green colors in the IPF maps correspond to the <001>, <111>, and <101> directions, respectively (Figure 5a–d). As the rolling reduction ratio increased, the deformed structure became gradually parallel to the rolling direction (Figure 3d and Figure 5d). The <110> // RD orientation was relatively strong under a rolling reduction of 80%. The grains were gradually distorted and fragmented into nano-scale grains. The textural evolution of UCBS in the φ2 = 45° section of the ODF is presented in Figure 5a1–d1. The undeformed sample exhibited a weak ζ-fiber texture ({011} <211>) (Figure 5a1), which became stronger with increasing the rolling ratio (Figure 5b1–d1). A strong {111} <110> γ texture was observed after 80% rolling reduction ratio.

**Figure 6:**
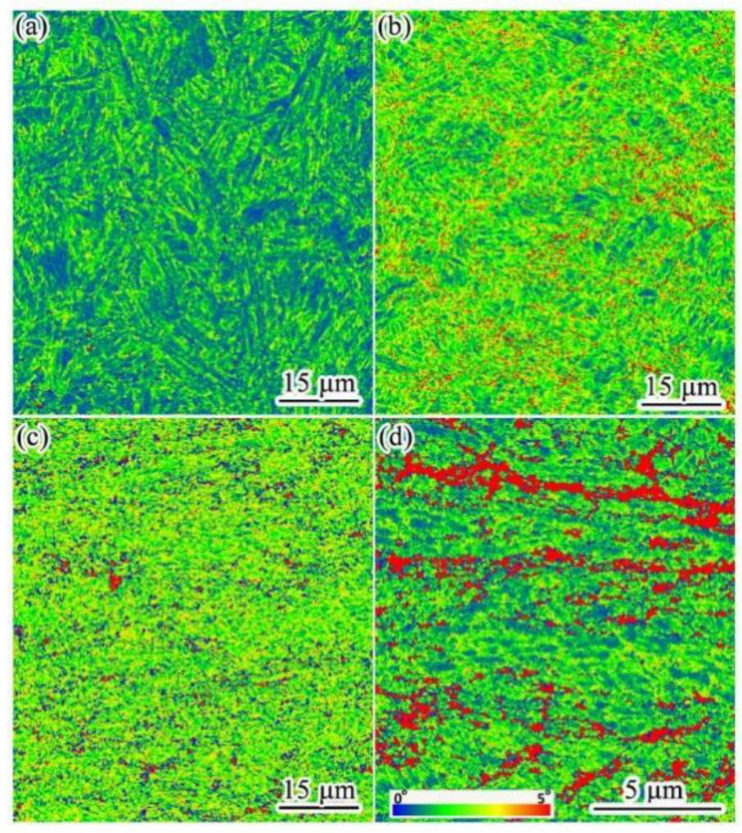
KAM maps of the UCBS. (**a**) Undeformed; (**b**) 20%, (**c**) 50%, and (**d**) 80% rolling reduction ratio.

Figure 6 presents the kernel average misorientation (KAM) distribution maps of UCBS obtained under different rolling reduction ratios. KAM provides the orientation gradients (from 0° to 5°) within individual grains, indicating the strain distribution caused by deformation. In general, the more uneven the strain distribution, the larger the strain gradient. According to the KAM maps, the wide regions of low KAM values (blue regions) indicate that the dislocation density was remarkably low, corresponding to the coarse laths in the undeformed sample (Figure 6a). The regions with high KAM values (red regions) are observed in Figure 6b–d, indicating that the lath/block boundary density increased as the reduction ratio increased to 80%. Nevertheless, some low KAM regions remained even after the 80% rolling reduction ratio.

**Figure 7:**
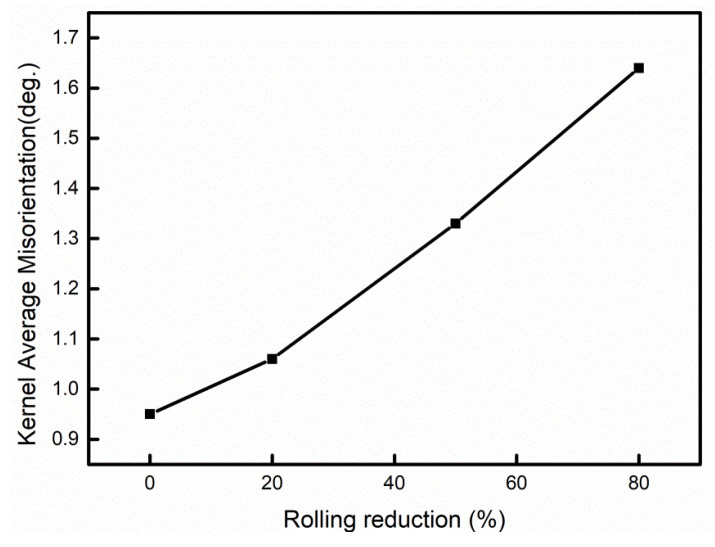
Variation of the average KAM values in different cold rolling reductions.

Figure 7 presents the average KAM angles of the UCBS on varying rolling reductions up to 80%. The average KAM angles are in the range of ~0.95° before rolling. As rolling reduction increased, the average KAM angle was greatly increased to 1.06° (20%) with a smaller augmentation reaching 1.33° at the reduction ratio of 50%. The average KAM angle (1.64°) at the 80% rolling reduction was higher. It was indicated that the dislocation density in bainite was remarkably increased between the 20% and 80% reduction ratios.

### 3.4. XRD Characterization

**Figure 8:**
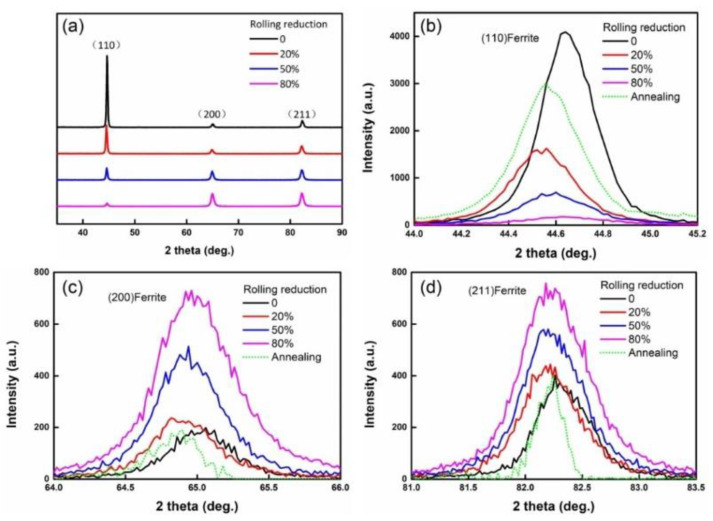
(**a**) X-ray diffraction patterns of UCBS under different rolling reductions, and X-ray diffraction pattern of the (**b**) (110), (**c**) (200), and (**d**) (211) peaks.

The XRD diffraction patterns of UCBS under different rolling reduction ratios are presented in Figure 8. Only ferrite could be identified in the different rolling reduction samples, due to the texture formed during the cold rolling. With increasing rolling reduction ratio, the (110) peak became weaker, and the (200) and (211) peaks became stronger, indicating that a preferred orientation of (200) and (211) peaks in the grains were formed after rolling (Figure 8a). Furthermore, the diffraction angle of the (110), (200), and (211) peaks first decreased under a 20% rolling reduction ratio. Due to the stress introduced by cold rolling, they then increased to 80% (Figure 8b–d).

## 4. Discussion

### 4.1. Effect of Microstructure Evolution on Mechanical Properties

In general, grain refinement can simultaneously improve the strength and elongation of steels, suggesting that microstructure refinement may also play a similar role [24,30,34,35]. During the rolling process, the amount of MA decreased with increasing rolling reduction ratio, which may be attributed to the partial transformation of austenite into martensite under the action of stress [36,37]. The width of the laths decreases with the cold rolling reduction ratio increasing (Figure 4). The revised Hall–Petch equation can be used to reveal the relationship between yield strength and microstructure. Since the propagation and slippage of dislocations occur mainly in the lath, the actual grain size can be substituted by using the lath thickness multiplied by 2 [38,39,40,41]:

(1) $$\sigma \left(y\right)=\sigma_{0}+k{\left(2d\right)}^{-0.5}$$

where *σ(y)* corresponds to the yield strength, *σ*_0_ corresponds to the friction stress of the ferrite and is a constant ~60 MPa, *k* is the slope, and *d* denotes the width of the laths (Table 1).

**Figure 9:**
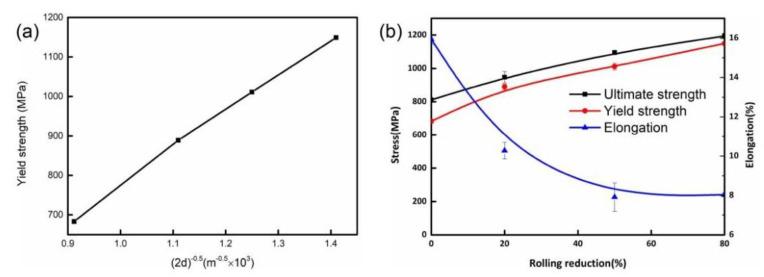
(**a**) Relationship between yield strength (σy), and the reciprocal of the square root of twice the width of ferrite lamellae (2d)^−0.5^; (**b**) mechanical properties under different rolling reduction ratios.

Based on the above calculations, the relationship between yield strength *σ(y)* and the width of the laths at different rolling reductions are shown in Figure 9. At a 20% rolling reduction ratio, *K* was about 0.68 MPa∙m^−0.5^. When the rolling reduction ratio exceeded 50%, the value of *K* remained approximately the same (0.75–0.77 MPa∙m^−0.5^) (Table 1). This indicates that, at the initial reduction, the main contribution to the increase in yield strength comes from the boundary strengthening mechanism. Then, the value of *K* indicates that, as the amount of reduction increases, the boundary strengthening contribution decreases, and the contribution of other strengthening mechanisms increase, such as dislocation strengthening [26]. Interestingly, at 80% reduction, the strength increases, and the elongation increases slightly. The grains are fragmented and elongated to form an ultra-fine grain structure under high strain (Figure 5d), which affects both strength and elongation [42,43,44,45]. At the same time, the formation of a strong {111} <110> texture has also a certain contribution to elongation [13,46].

### 4.2. Effect of Dislocation Density Evolution on Mechanical Properties

In addition to refining the microstructure, dislocation density also significantly affects the mechanical proprieties [20,47,48]. As the rolling reduction ratios increase, the dislocation density increases significantly, as observed in the TEM images and KAM maps (Figure 4, Figure 6, and Figure 7, respectively). In order to quantitatively analyze the variation of dislocation density under different rolling reductions, the modified Williamson–Hall equation (W–H) can be employed, which can be expressed in Equation (2) [17,47,49,50]:

(2) $$\Delta K≅\frac{1}{d}+\left(\frac{\pi M^{2}b^{2}}{2}\right)\rho^{1/2}\left(K^{2}\bar{C}\right)+O\left(K^{4}\bar{C^{2}}\right)$$

where *K* = 2 sin θ/λ; △*k =*2 cos *θ(*△*θ)/λ*; *θ* and △*θ* are diffraction angle and the integral breadth of the diffraction peak (removed instrument broadening at half-width), respectively; *λ* is the wavelength of the X-rays; *D* is the average grain size; *b* is the Burgers vector; *ρ* is the average dislocation density; *M* is a constant related to both effective outer cut-off radius of the dislocations and the dislocation density. In general, a value of *M* = 2 is suitable for deformed materials [47,49]. In addition, *K*^2^*C* represents the average contrast factor of dislocations, and *O* indicates non-interpreted higher-order terms in *K*^4^*C*^2^. However, the term that contains it in Equation (2) is ignored [49].

**Figure 10:**
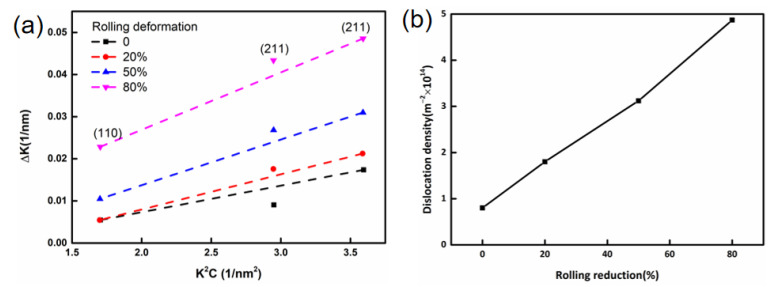
(**a**) Dislocation density of UCBS under different cold rolling reductions and (**b**) modified W–H plot of the integral breadth of the diffraction peaks.

Three peaks, i.e., (110), (200), and (211), were recorded for each line profile. The dislocation density can be calculated by the slope obtained by fitting the curve *ρ* = 2 m^2^/(πM^2^b^2^), where *m* is the slope of the line. The modified W–H plots and dislocation density under all conditions are demonstrated in Figure 10. It can be observed that, as the rolling reduction ratio increased to 20%, the dislocation density increased from 8.3 × 10^13^ m^−2^ to 1.8 × 10^14^ m^−2^, the dislocation strengthening was not apparent, while the boundary strengthening (fine grain strengthening) was dominant, which is in accordance with the KAM maps. When the rolling reduction ratio increased from 50% to 80%, the dislocation density increased significantly from 8.3 × 10^13^ m^−2^ to 1.8 × 10^14^ m^−2^, and the dislocation strengthening effect became stronger.

### 4.3. Texture Analysis

In ferritic steels (BCC crystals), the {110} crystallographic plane is the desired plane that can improve the toughness [34]. The texture of BCC steels during cold rolling has been previously investigated [46,51,52]. In general, the {001} <110> component is the most stable texture. With an increasing rolling reduction ratio, the crystals of the grains rotate gradually towards the <110> direction, which is parallel to the direction of deformation [13,53]. At the rolling reduction ratios of 20% and 50%, the ζ-fiber texture ({011} <211>) becomes stronger. When the deformation increases to 80%, a γ-fiber {111} <110> texture appears, where the deformation takes place slowly in low carbon steel [54]. A larger number of boundaries prevent dislocation movement [19], which is why the elongation did not decrease significantly, compared to that under the 50% rolling reduction ratio. Consequently, the ζ-fiber texture of undeformed steel was substituted by γ-fiber texture when the rolling reduction ratios were increased to 80%.

### 4.4. Strength Analysis

In the rolling process, three strengthening mechanisms relate microstructure to strength, i.e., boundary strengthening σ(*b*), solution strengthening σ(*s*), and dislocation strengthening σ(*ρ*), which were analyzed. It is assumed that both strength contributions during the rolling process are additive. Thus, the strength can be expressed as Equation (3) [38,39].

(3) σ(ε)=σ(s)+σ(b)+σ(ρ)

Boundary strengthening can be represented by Equation (4) [21].

(4) $$\sigma \left(b\right)=K_{b}{\left(2d\right)}^{-0.5}$$

In general, it is difficult to estimate the value of *k* with the increasing rolling stress based on the experimental results. In order to facilitate this calculation, the Hall–Petch relationship is employed with $k_{b}$ = 0.68.

Dislocation strengthening is based on forest hardening, which was expressed as Equation (5) [39].

(5) $$\sigma \left(\rho \right)=M\alpha Gb\sqrt{\rho }$$

where *M* is the orientation factor (same as Equation (2)); *a* = 0.2 is a constant; *G* = 77.5 GPa is the shear modulus of ferrite; *b* is the Burgers vector; *ρ* is the dislocation density calculated earlier. The calculated values of *ρ* are given in Figure 9.

**Figure 11:**
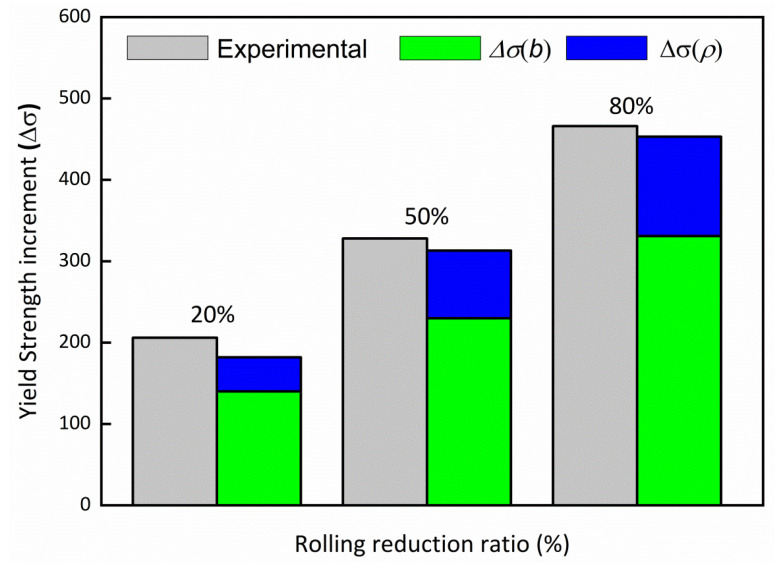
Schematic diagram of yield strength increment under different cold rolling reductions. Experimental results (gray), theoretical contribution of boundary strengthening (green), and dislocation strengthening (blue).

The effect of solution strengthening on strength increment can be ignored because there is no significant change during the cold rolling process. The experimental yield strength increment and contributions of boundary strengthening and dislocation strengthening are shown in Figure 11. Based on the above calculations, the trend of theoretical strength agrees with the experimental trend under different cold rolling reductions. However, the strength values experimentally determined were higher than the theoretical values since the strengthening of the small amount of martensite (transformed from filmy retained austenite) were not considered in the calculations [36]. Thus, the strength increment can be mainly attributed to the two strengthening mechanisms.

## 5. Conclusions

In this paper, the microstructure and mechanical properties development of high strength UCBS under different cold rolling reductions was investigated. The following conclusions can be drawn from the obtained results.

(1) With the cold rolling reduction increased to 80%, the microstructure was refined, and the lath width decreased from 601 nm to 252 nm. The UTS and YS increased from 812 MPa and 683 MPa to 1195 MPa and 1150 MPa, respectively, while the elongation decreased from to 15.9% to 7.9%.
(2) With cold rolling reduction ratios increasing, the local stress distribution was not significantly more uniform. The dislocation density increased from 8.3 × 10^13^ m^−2^ to 4.87 × 10^14^ m^−2^ and a stronger γ-fiber texture was obtained at the 80% cold rolling reduction ratio.
(3) The yield strength of UCBS increased mainly due to boundary strengthening and dislocation strengthening. The theoretical yield strength increment is based on the two strengthening mechanisms which agree with the experimental measurements.

## Figures and Tables

**Table 1:** Slope *k* of the Hall–Petch relationship under different cold rolling reduction ratios.

Rolling Reduction Ratio (%)	0	20	50	80
*d* (nm)	601	404	320	252
$\sigma y-\;\sigma_{0}$ (MPa)	623	829	951	1089
*k*	0.68	0.75	0.76	0.77

## Data Availability

No new data were created or analyzed in this study. Data sharing is not applicable to this article.
